# Global and Regional Structural Differences and Prediction of Autistic Traits during Adolescence

**DOI:** 10.3390/brainsci12091187

**Published:** 2022-09-02

**Authors:** Frauke Nees, Tobias Banaschewski, Arun L. W. Bokde, Sylvane Desrivières, Antoine Grigis, Hugh Garavan, Penny Gowland, Yvonne Grimmer, Andreas Heinz, Rüdiger Brühl, Corinna Isensee, Andreas Becker, Jean-Luc Martinot, Marie-Laure Paillère Martinot, Eric Artiges, Dimitri Papadopoulos Orfanos, Hervé Lemaître, Argyris Stringaris, Betteke van Noort, Tomáš Paus, Jani Penttilä, Sabina Millenet, Juliane H. Fröhner, Michael N. Smolka, Henrik Walter, Robert Whelan, Gunter Schumann, Luise Poustka, on behalf of the IMAGEN Consortium

**Affiliations:** 1 Institute of Medical Psychology and Medical Sociology, University Medical Center Schleswig Holstein, Kiel University, 24118 Kiel, Germany; 2 Department of Child and Adolescent Psychiatry and Psychotherapy, Central Institute of Mental Health, Medical Faculty Mannheim, Heidelberg University, Square J5, 68159 Mannheim, Germany; 3 Institute of Cognitive and Clinical Neuroscience, Central Institute of Mental Health, Medical Faculty Mannheim, Heidelberg University, Square J5, 68159 Mannheim, Germany; 4 Discipline of Psychiatry, School of Medicine and Trinity College Institute of Neuroscience, Trinity College Dublin, Dublin 2, Ireland; 5 Institute of Psychiatry, Psychology & Neuroscience, Centre for Population Neuroscience and Precision Medicine (PONS), SGDP Centre, King’s College London, London WC2R 2LS, UK; 6 NeuroSpin, CEA, Université Paris-Saclay, 91191 Gif-sur-Yvette, France; 7 Departments of Psychiatry and Psychology, University of Vermont, Burlington, VT 05405, USA; 8 Sir Peter Mansfield Imaging Centre School of Physics and Astronomy, University of Nottingham, University Park, Nottingham NG7 2QL, UK; 9 Department of Psychiatry and Psychotherapy CCM and Berlin Institute of Health, Charité—Universitätsmedizin Berlin, Corporate Member of Freie Universität Berlin, Humboldt-Universität zu Berlin, 10117 Berlin, Germany; 10 Physikalisch-Technische Bundesanstalt (PTB), 38116 Braunschweig, Germany; 11 Department of Child and Adolescent Psychiatry and Psychotherapy, University Medical Centre Göttingen, von-Siebold-Str. 5, 37075 Göttingen, Germany; 12 Institut National de la Santé et de la Recherche Médicale, INSERM U A10 “Trajectoires Développementales en Psychiatrie”, Université Paris-Saclay, Ecole Normale Supérieure Paris-Saclay, CNRS, Centre Borelli, 91190 Gif-sur-Yvette, France; 13 Department of Child and Adolescent Psychiatry, Pitié-Salpêtrière Hospital, AP-HP Sorbonne Université, 75013 Paris, France; 14 Psychiatry Department, EPS Barthélémy Durand, Gif-sur-Yvette, 91150 Etampes, France; 15 Institut des Maladies Neurodégénératives, UMR 5293, CNRS, CEA, Université de Bordeaux, 33076 Bordeaux, France; 16 National Institute of Mental Health/NIH, 15K North Drive, Bethesda, MD 20892, USA; 17 MSB Medical School Berlin, Hochschule für Gesundheit und Medizin, Siemens Villa, 14197 Berlin, Germany; 18 Departments of Psychology, University of Toronto, Toronto, ON M5T 2S8, Canada; 19 Department of Psychiatry, University of Toronto, Toronto, ON M5T 2S8, Canada; 20 CanadaDepartment of Social and Health Care, Psychosocial Services Adolescent Outpatient Clinic Kauppakatu 14, 15140 Lahti, Finland; 21 Department of Psychiatry, Neuroimaging Center, Technische Universität Dresden, 01069 Dresden, Germany; 22 School of Psychology, Global Brain Health Institute, Trinity College Dublin, Dublin 2, Ireland; 23 PONS Research Group, Department of Psychiatry and Psychotherapy, Campus Charite Mitte, Humboldt University, 10117 Berlin, Germany; 24 Leibniz Institute for Neurobiology, 39118 Magdeburg, Germany; 25 Institute for Science and Technology of Brain-inspired Intelligence (ISTBI), Fudan University, Shanghai 200437, China

**Keywords:** adolescents, autism spectrum disorder, autistic traits, social responsiveness, structural imaging

## Abstract

Background: Autistic traits are commonly viewed as dimensional in nature, and as continuously distributed in the general population. In this respect, the identification of predictive values of markers such as subtle autism-related alterations in brain morphology for parameter values of autistic traits could increase our understanding of this dimensional occasion. However, currently, very little is known about how these traits correspond to alterations in brain morphology in typically developing individuals, particularly during a time period where changes due to brain development processes do not provide a bias. Therefore, in the present study, we analyzed brain volume, cortical thickness (CT) and surface area (SA) in a cohort of 14–15-year-old adolescents (N = 285, female: N = 162) and tested their predictive value for autistic traits, assessed with the social responsiveness scale (SRS) two years later at the age of 16–17 years, using a regression-based approach. We found that autistic traits were significantly predicted by volumetric changes in the amygdala (*r* = 0.181), cerebellum (*r* = 0.128) and hippocampus (*r* = −0.181, *r* = −0.203), both in boys and girls. Moreover, the CT of the superior frontal region was negatively correlated (*r* = −0.144) with SRS scores. Furthermore, we observed a significant association between the SRS total score and smaller left putamen volume, specifically in boys (*r* = −0.217), but not in girls. Our findings suggest that neural correlates of autistic traits also seem to lie on a continuum in the general population, are determined by limbic–striatal neuroanatomical brain areas, and are partly dependent on sex. As we imaged adolescents from a large population-based cohort within a small age range, these data may help to increase the understanding of autistic-like occasions in otherwise typically developing individuals.

## 1. Introduction

Autistic traits are defined as mild, subclinical deficits in social interaction and communication, as well as restricted behaviors, interests and activities that are continuously distributed in the general population. There is convincing evidence that these traits lie on a continuum, with autism spectrum disorder (ASD) as the extreme end of a normal distribution of quantitative characteristics [1]. Like ASD itself, such autistic traits suggest a high heritability, ranging from 36–87% (e.g., [2]). At the same time, findings from neurobiological research strongly suggest structural changes in the brain, including changes in the cerebellum, hippocampus, amygdala or caudate, underlie the characteristic symptomatology in ASD (e.g., [3,4]). However, results are controversial. The largest study on ASD-specific brain changes in individuals with ASD vs. typically developed (TD) controls (*n* = 1571), Enhancing NeuroImaging Genetics through Meta-Analysis (ENIGMA) ASD [3], showed ASD-related increased thickness in the frontal cortex and smaller subcortical volumes, e.g., of the amygdala and the putamen, with a clear developmental peak round adolescence [3]. Moreover, one also needs to consider whether findings provide association patterns for one time point or prediction analyses from brain structure to symptoms and/or vice versa. Longitudinal studies on brain development in ASD suggest that brain morphological differences between individuals with ASD and TD controls show a high variability across different developmental periods, with early brain overgrowth in childhood being followed by attenuated or slowed growth in later childhood and significant volume decrease during adolescence and adulthood [5].

Findings also differ depending on the different magnetic resonance imaging (MRI) techniques for brain morphology. Sussman and co-workers [4] comprehensively investigated these neuroanatomical parameters in a cohort of children and adolescents with ASD (*n* = 72) aged between 4–18 years. They found widespread changes in the cortical, subcortical and cerebellar regions in participants with ASD as compared to TD controls, which is in accordance with the literature on brain growth trajectories in ASD (e.g., [6]). Additionally, they observed a lack of age appropriate cortical thinning, specifically in the orbitofrontal cortex and the left posterior cingulate, together with an age-independent decreased volume of the left hippocampus in the ASD groups [4].

Interestingly, attenuated abnormalities in brain structure have not only been observed in clinical populations of ASD, but also in non-affected siblings of individuals with ASD (e.g., [7]). In support of this observation, brain morphological correlates of ASD should also be present to a lesser degree in the general population. The investigation of neurobiological characteristics in relation to autistic traits in populations of TD individuals therefore represents a valid and promising approach for our understanding of endophenotypes of the condition. In this respect, prediction analyses on the effects of brain structure on the parameter values of autistic traits, and during a time period where changes due to brain development processes do not provide a bias, might be most informative. However, to date, only a few studies have investigated the association of brain morphology and autistic traits in population-based samples, and mainly in a cross-sectional fashion. In typically developed adults, smaller volumes in the inferior parietal lobule [8] and insula [9] in relation to ASD-symptomatology have been reported. Wallace et al. [10] found increased cortical thickness (CT) in the bilateral middle and superior temporal region to correlate with autistic traits in children and young adults, and Tu et al. [11] reported reduced CT in the insula and the right superior temporal gyrus in a sample of healthy male adolescents. Blanken and co-workers [12] examined a large sample of typically developing children between 6–10 years, and found associations between autistic traits and a widespread decrease of cortical gyrification. When children with the highest scores of autistic traits were excluded from the analysis, a strong linear relationship remained between autistic symptoms and the left temporal and precuneus area of the left hemisphere. Thus, although there is evidence on associations between autistic traits and brain structure in healthy populations, observed relationships are also somewhat inconsistent; some studies did not find any significant associations between autistic traits and brain morphometry (e.g., [13]), or do not converge with reports from studies in ASD (e.g., [14]). These inconsistencies could have several reasons: (a) the assessment of autistic traits, which range from means of the Social Responsiveness Scale (SRS, [15]) to the Autism Quotient (AQ, [16]); (b) the study design, which was, so far, of a cross-sectional nature, and thus, did not provide any direct evidence on how brain morphology confers risk to ASD-related traits during specific developmental periods; and (c) the age range, which was rather wide in previous studies, between 6–16 years, and where general morphological differences during development could have co-affected the results [14].

### Main Aim of the Study

Together, autistic traits are commonly viewed as dimensional in nature, and as continuously distributed in the general population. However, currently, very little is known about how these traits correspond to alterations in brain morphology in typically developing individuals, particularly during a time period where changes due to brain development processes do not provide a bias in terms of predictive effects. Therefore, in the present study, we aimed to analyze data from a longitudinal study with a very well characterized, large, age-homogenous sample of typically developed adolescents. We hypothesized that distributions in brain structure are significant predictors of the distribution of autistic traits in TD. For brain morphology, we used measures of cortical volume (CV) and CT at the age of 14–15 years, and used SRS as the outcome measure two years later at the age of 16–17 years. While CV does not reflect cortical organization [17], CT, as a parameter of CV, provides rather sufficient information on the organizational processes of cortical neurons. Since previous studies found substantial sex differences regarding brain morphology in a sample of individuals with ASD (e.g., [4]), we also tested for possible differences between boys and girls in our TD sample.

## 2. Materials and Methods

### 2.1. Participants

We used data from healthy adolescents (N = 285; see Table 1 for sample) of the longitudinal Imaging Genetics (IMAGEN) study who were assessed at the age of 14–15 years, and followed-up two years later at the age of 16–17 years.

IMAGEN is a large European multicenter study that aimed at investigating how biological, psychological and environmental factors during adolescence may influence brain development and mental health. Using brain imaging and genetics, the project will help develop prevention strategies and improved therapies for mental health disorders in the future. Study participation included ‘home assessments’, which comprised cognitive and neuropsychological tests through a web-based coordinated system (Psytools, see below), and 1–2 study centre visits, where adolescents underwent brain imaging, blood sampling and diagnostic interviews, and parents were also interviewed and filled out some questionnaires, with both questions that targeted themselves as well as questions about their children.

Adolescents were recruited from the general public via school visits, flyers and registration offices in Germany, Ireland, France and the United Kingdom. Exclusion criteria were serious medical conditions, the presence of any mental disorder as defined by the Development and Well-Being Assessment (DAWBA; [18]), based on clinical ratings of the interview data, the presence of ASD diagnosis, and contra indications for MRI exams including pregnancy, metal parts in the body, and previous head trauma with unconsciousness. We did not obtain any information on medication status nor on whether participants had siblings with an attention deficit hyperactivity disorder (ADHD) diagnosis. The study was approved by the local ethics committees and adhered to the Declaration of Helsinki. Written informed consent from both the adolescents and their parents was obtained after complete description of the study.

### 2.2. Screening for Intelligence

We estimated general intelligence using Block Design, Similarities, Vocabulary and Matrix Reasoning of the Wechsler Intelligence Scale for Children, Version IV (WISC-IV; [19]). Two indices were calculated from the WISC subtests—on the one hand, the index Verbal Comprehension derived from the subtests Vocabulary and Similarities, on the other hand, the index Perceptual Reasoning derived from the subtests Block Design and Matrix Reasoning.

### 2.3. Autistic Traits: Social Responsiveness Scale

To measure autistic traits, we used the Social Responsiveness Scale (SRS; see [15]), a well-validated parent/teacher-report to assess deficits in reciprocal social behavior as a continuous variable in 4- to 18-years-olds. For the present study, parents filled out the questionnaire and we used the total SRS score, representing an index of impairment in social communication and interaction, which could be computed. The French SRS data were excluded from the analysis, because there is no validation of the French SRS version available. For the assessment of the SRS, we used the behavioural characterization Psytools software (Delosis Ltd., London, UK) via its internet-based platform.

### 2.4. Magnetic Resonance Imaging

Brain data were obtained using a 3T whole body MRI system, equipped with a standard 12-channel head coil, made by several manufacturers (Siemens, Philips, General Electric, Bruker) at the eight IMAGEN assessment sites (Paris, London, Dublin, Nottingham, Dresden, Berlin, Mannheim and Hamburg). We applied image-acquisition techniques using a set of parameters compatible with all scanners that were implemented to ensure a comparison of the MRI results. Structural T_1_-weighted images were acquired using a 3D magnetization prepared gradient echo sequence (MPRAGE) based on the Alzheimer`s Disease Neuroimaging Initiative (ADNI) protocol (repetition time (TR) = 6.8 ms and echo time (TE) = 3.2 ms; flip angle = 8° 256 × 256 × 160 matrix; voxel size: 1.1 × 1.1 × 1.1 mm) covering the whole brain.

Analyses were done centrally at one location, where data for the central database were managed and processed, and in addition, segmentation was also manually controlled as part of the central quality control procedures. We analyzed the CV and CT of designated regions of interest (ROIs) using the FreeSurfer software. This is a set of automated tools for reconstruction of brain cortical surface [20], which included cortical and subcortical volumetric segmentation, cortical grey matter and white matter surface reconstruction [20] and the estimation of total intracranial volume (ICV) [21] applying Talairach transformation, intensity normalization and removal of non-brain tissue. We used the structural images to segment white and gray matter and estimated the gray–white matter interface, which served as reference for a deformable surface algorithm searching for the pial surface. Deformable procedures included surface inflation, creation of a variety of surface-based data (maps of curvature and sulcal depth) and registration to a spherical atlas, which is based on individual cortical folding patterns to match cortical geometry across individuals. To produce representations of cortical thickness, both intensity and continuity information from the entire three dimensional MR volume in segmentation and deformation procedures were used, and a calculation of the closest distance from the gray–white boundary to the gray–CSF boundary at each vertex on the tessellated surface was applied [22]. This was controlled for in a visual inspection (N = 8 individuals were excluded for the final analyses).

Cortical thickness was estimated based on the difference between the position of equivalent vertices in the pial surface and grey–white matter interface, while differences between the depth of gyri and sulci were normalized and the surface of the grey–white matter border was inflated. We morphed and reconstructed the brain of each adolescent to an average spherical surface and smoothed the data on the level of the sphere (Gaussian smoothing kernel with a full-width half maximum of 15 mm) to obtain difference maps of CT.

We performed analyses for the following individual regions of interest (ROIs) according to previous studies (e.g., [4,13]): amygdala, caudate nucleus, hippocampus, globus pallidum, nucleus accumbens, putamen, cerebellum and thalamus for brain volume, and frontal regions for cortical thickness.

### 2.5. Statistical Analyses

To explore the prediction of social responsiveness (16–17 years) by cortical volumes (14–15 years) of our ROIs, we performed regression analysis in the whole group, as well as separately for boys and girls, given the lack of gender norms for the SRS and general differences in brain volume between boys and girls. Moreover, to test whether associations are specific for a high-risk group, we performed an add-on analysis, where individuals with SRS scores above 50 (N = 29) were excluded from the regression analysis. This was based on Lyall et al. [23], where clinically ‘relevant’ scores were found at a lower level, around 50, on the SRS. We applied α = 0.05 and Bonferroni correction, using the Statistical Package of Social Sciences (IBM SPSS Statistics for Window, Version 20.0, BM Corp, Armonk, NY, USA). The effect of the MRI site was controlled by adding the study center as a nuisance covariate in the analysis, and we also controlled for total intracranial volume. 

## 3. Results

### 3.1. Sample Characteristics

We found significant differences between boys and girls regarding the social awareness subscale, but not for social cognition, social communication, social motivation and mannerism subscales, nor for the total SRS score (Table 1). An overview on the structural data can be found in Table 2.

### 3.2. Subcortical Brain Volume and the Prediction of Social Responsiveness

**Figure 1:**
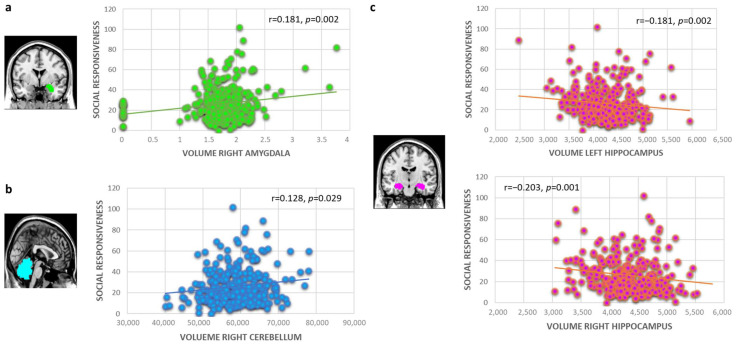
Prediction of social responsiveness by volumetric changes in (**a**) the right amygdala, (**b**) the right cerebellum, and (**c**) the left and right hippocampus.

We found significant associations between the SRS total score and larger right amygdala (*r* = 0.181, *p* = 0.002, explained variance (EV) = 3.7%; Figure 1a), larger right cerebellum (*r* = 0.128, *p* = 0.029, EV = 2.0%; Figure 1b) and smaller left and right hippocampus volumes (left: *r* = −0.181, *p* = 0.002, EV = 1.4%; right: *r* = −0.203, *p* = 0.001, EV = 2.5%; Figure 1c). In total, 11.7% of the variance was explained by the volume of these brain regions (*p* < 0.001; right amygdala: *t* = 4.273, *p* < 0.001; right cerebellum: *t* = 2.654, *p* = 0.008; left hippocampus: *t* = −1.949, *p* = 0.052; right hippocampus: *t* = −2.479, *p* = 0.014). Except for the hippocampus, these associations did not persist when excluding adolescents with an SRS total score higher than 50 (remaining sample: N = 259).

**Figure 2:**
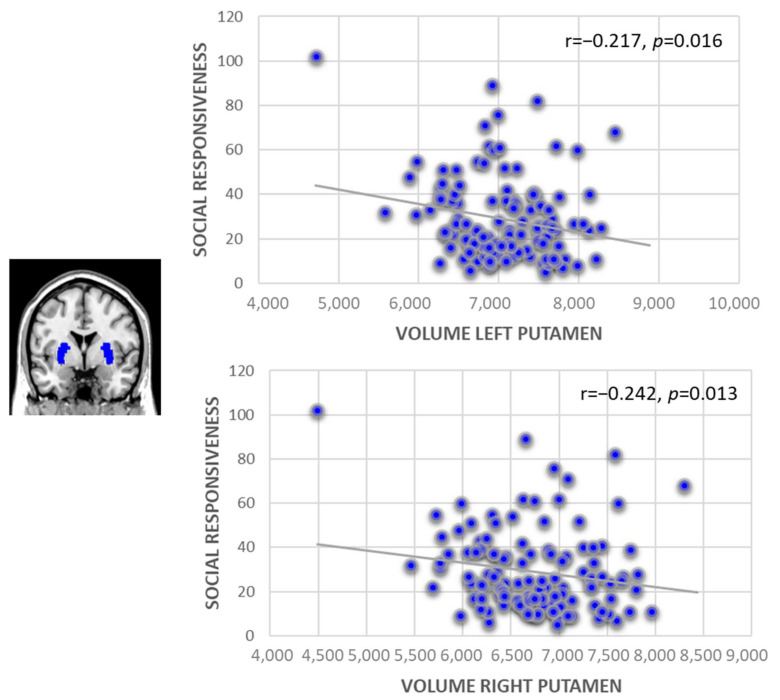
Prediction of social responsiveness by volumetric changes in the left and right putamen in male adolescents.

Moreover, we observed a significant association between the SRS total score and smaller left putamen volume in boys (*r* = −0.217, *p* = 0.016, EV = 4.4%; Figure 2), but not in girls. This finding persists also when excluding the group with high SRS scores, and expands to the right putamen (*r* = −0.242, *p* = 0.013, EV = 6.3%; Figure 2).

### 3.3. Cortical Thickness and the Prediction of Social Responsiveness

For CT, we found a significant negative correlation between the SRS total scale and the left superior frontal region (*r* = −0.141, *p* = 0.017, EV = 2.1%), which persists after exclusion of the sample with higher SRS scores.

## 4. Discussion

In the present study, we examined whether brain morphology correlates of autistic traits are present along a continuum in a population of typically developing adolescents, testing the predictive impact of CV and CT on autistic traits. Volumes of the cerebellum, amygdala and hippocampus at age 14–15 years were associated with autistic symptoms, as measured with the SRS 2 years later. Cerebellum and amygdala volumes were positively associated, and hippocampal volumes negatively associated, with SRS scores. CT of the superior frontal region was negatively associated with autistic traits 2 years later, while no predictive association was found between SA with SRS scores.

Since our aim was to specifically investigate a large TD, non-ASD sample, from the general population, we need to consider the SRS mean and distribution in our sample. The mean SRS total score was below a critical cut-off in terms of autism-related symptomatology. However, there is also a good range of values within the sample. We still need to note that speaking about autistic traits, therefore, does not refer to highly noticeable clinical problems.

### 4.1. Volumetric Findings

In our population of typically developing adolescents, sub threshold deficits in interaction and communication were preceded by brain morphological characteristics in some of those brain areas, which are discussed as functionally related to abilities that are impaired in ASD. This applies to limbic brain regions such as the amygdala–hippocampal complex and the cerebellum, as well as the putamen.

In order to explore whether these associations in our study were mainly driven by participants with higher than average levels of autistic traits, we excluded adolescents with social responsiveness scores >50. The correlation between brain volume and autistic traits did not hold for the right amygdala and the cerebellum, but remained significant in the left and right hippocampus. This reveals a robust correspondence of autistic traits with reduced brain volume in an area related to social processing, contextual embedding and memory, and spatial learning. Anderson et al. [24] delineated in an extensive review on electrophysiological animal experiments the essential role of the hippocampus for behaviors associated with contextual novelty; one might speculate that individuals with low novelty seeking behavior, as is observed in ASD (e.g., [25]), will possibly show a decrease of volume through less functional demand on that structure. This might be due to a decline in the number of synaptic receptors [26], an observation that stems from findings for the stratum radiatum of a mouse model of ASD [26]. Our results are in line with the findings from Sussmann and co-workers [4] in a large group of adolescents between 12–18 years with ASD (*n* = 72), who had decreased hippocampal volumes in comparison to age-matched controls (*n* = 138).

However, as with other brain areas, previous volumetric findings regarding the hippocampus in ASD showed an inconsistent pattern—both larger (e.g., [27]) and lower (e.g., [4]) volumes have been reported in individuals with ASD compared to typically developing controls, and some studies also found no volumetric difference [28]. A hypothesis explaining the enlargement of the hippocampus–amygdala complex in ASD (e.g., [27]) suggests an increase of hippocampal volume in response to heightened amygdala activity due to chronic stress in ASD, as the hippocampus exerts a regulatory effect on the amygdala via multiple reciprocal connections.

Both these theories seem logical, while reflecting characteristic behavioral variations in ASD, and need further verification. The so far inconsistent findings may possibly be related to the heterogeneity of the investigated samples, including the developmental effects. As our analysis was performed in typically developing adolescents within a very small age range are, the results significantly contribute to the existing literature by reducing the effects of age and developmental stage.

At the same time, we observed positive associations between autistic traits and cerebellar volume. The cerebellum is a crucial structure for the initial stages of learning and an important node in the cortico-cerebello-thalamo-cortical circuit. The latter is considered to play a key role in the pathophysiology of autism; early disruptions in these circuits may impact the development of language, social cognition, attention and literacy acquisition [29]. This might first inspire assumptions regarding a reduction of cerebellar volumes in ASD. However, very recent findings by Pote et al. [30] point towards significantly larger cerebellar volumes in high-risk infants (later-born siblings of children with ASD) at 4–6 months. The enlargement was related to more repetitive behaviors, also a core domain in ASD at age 36 months, which is in line with our findings on increased cerebellar volumes and higher autistic traits. As both studies were done in non-clinical samples and at critical developmental stages, findings suggest that volumetric changes can function as predictive biomarkers for autistic traits in childhood and adolescence. The fact that the association between cerebellar volumes and autistic traits did not remain significant after exclusion of the adolescents who scored at the high and clinically relevant trait levels corroborates these assumptions.

After excluding those with the highest levels of autistic traits, we also found that smaller volumes of the putamen, as part of the striatum, significantly predicted more problems in social responsiveness, but only in boys. The role of the putamen includes the representation of action-specific values that are important for the mediation between reward and adaptive behavior [31]. The putamen is involved in the linking of reward to action and in planning actions (e.g., [32]), including a propensity to engage in goal-directed over habitual behaviors. These behaviors might be reduced in boys compared to girls. On the other hand, we observed significantly higher SRS scores in boys for the subscales of social communication and social awareness, indicating higher impairment in these domains compared to girls. Furthermore, diffusion tensor imaging studies by Langen and colleagues [33] have shown decreased white matter connectivity between the striatum and the prefrontal cortex in ASD compared with typically developed individuals. These findings suggest that the putamen abnormality observed in ASD might be related not only to the domain of motor/repetitive behaviors per se, but also to broader ASD phenotypes, such as impairments in higher cognitive functioning, including emotional facial recognition and reciprocal social interaction, as measured with the SRS.

### 4.2. Cortical Thickness and SRS

We also examined two subcomponents of cortical volume, CT and SA, and found negative associations between autistic traits and CT of the superior frontal regions, and no differences in SA. This is not in line with studies in clinical samples. Results by Sussmann et al. [4] and van Rooj et al. [5], in samples with participants with ASD and TD controls, showed a cross-sectional increase of CT in the frontal brain areas. The superior frontal cortex is related to the control of behavior and cognition, including self-awareness and decision making (e.g., [34]). Reduced volume in the frontal cortex might, therefore, be a correlate for a loss of controlling (adequate) behaviors, including those regarding social situations and contact, and adolescents with higher autistic traits might have lower behavioral control.

### 4.3. Autistic Traits and Brain Development

As mentioned above, brain–behavior correlates during childhood and adolescence must always be seen in the light of developmental influences and differences. One has to bear in mind that neuroanatomical alterations in ASD are not confined to single brain regions, but rather reflect disturbances of wider neural systems and networks (e.g., [35]). Neuroanatomical differences between individuals with ASD and TD controls are highly variable across different stages of development, and a significant increase of brain volume during early development might be associated with a significant decrease of volume during adolescence and adulthood.

Looking at growth trajectories in ASD over time, volumetric abnormalities of e.g., the cerebellum are consistently found in morphometric studies of ASD populations, mainly in respect to overall increase in children (e.g., [36]) and adolescents [4]. Differences are mostly driven by white matter increase and seem to taper off in late adolescence [37], which is possibly the reason why our results did not hold when participants with the highest SRS scores were excluded from the analysis. However, Hardan et al. [36] reported on enlarged cerebellar volumes in high functioning young adults with ASD. Other investigations observed significant correlations between measures of social and repetitive behavior and increased volume of striatal regions, and decreased volume in multiple frontal and temporal regions and the cerebellum in children and adults with ASD (e.g., [38]). Gray matter reductions in the cerebellum were significantly associated with deficits in integrating and regulating cognitive and emotional functions in children with ASD [39] and with scores on the Autism Diagnostic Interview (ADI-R), which were additionally shown to be associated with increased thickness in the parietal and frontal regions in adults with ASD [40]. These findings already indicate partly controversial effects, and thus, a neuroanatomical autism “puzzle” (e.g., [4]), as also found for other disorders like for obsessive-compulsive disorder, as a clinically heterogeneous disorder of symptom dimensions. As we analyzed data from adolescents within a very small age range, and performed prediction but not cross-sectional correlation analyses of autistic traits, our findings add potential correlates dedicated to a specific life period and markers that might serve as indicators for future behaviors to the existing literature, including processes of social functioning. The findings also underline the importance of further unraveling interactions and clinical symptomatology, even if, or specifically since, our findings stem from TD individuals with mean SRS scores below a clinically relevant cut-off. As trait-related deficits and behavioral ASD symptoms change over time, linked by neurobiological underpinnings, it is necessary to further gain insights into the developmental stages of the disorder.

### 4.4. Strengths and Limitations

Our present study possesses several strengths that may help to explain apparent discrepancies when comparing our findings with previous longitudinal imaging studies (e.g., [5]). We imaged adolescents from a large population-based cohort within a small age range and with autistic traits across a broad spectrum. This provides the unique opportunity to test whether the underlying brain morphology of autistic traits extends into the general population. In addition, considerable information is available on potential confounding factors. However, we also need to consider the mainly observed earlier age of onset of ASD when interpreting the findings from the present study, along with possible clinical implications. In this respect, we also need to take into account that we do not have SRS data from when the adolescents were 14 years old, and thus, from the time when brain imaging had been applied; therefore, we do not know whether the associations found for our prediction analyses were already present when the adolescents were 14 years old.

### 4.5. Conclusions

In the present study, we investigated the predictive value of brain morphology in TD for the continuous distribution of autistic traits. Brain imaging has often been applied in ASD to describe functioning and to identify potential risk markers (e.g., [41,42]). We found that autistic traits correspond to subtle autism-related alterations in brain morphology in typically developing individuals, as well as lying on a continuum in the general adolescent population, even at levels below a clinically revenant cut-off. This may increase our understanding of the underlying neurobiological processes for maladaptive social behavior. Future studies could link this information with further autism-related aspects of social processing [43,44].

## Figures and Tables

**Table 1:** Overview on demographic, intelligence and autistic trait measures.

	Total (N = 285) M (SD), Range	Male (N = 123) M (SD), Range	Female (N = 162) M (SD), Range	Significance Male vs. Female
WISC Verbal Comprehension	110.21 (13.56)	110.18 (14.93)	110.22 (14.98)	n.s.
WISC Reasoning	107.33 (14.19)	108.64 (14.22)	106.83 (15.13)	n.s.
SRS social communication	17.25 (4.15), 5–39	16.97 (4.31), 10–36	17.46 (4.03), 5–39	n.s.
SRS social awareness	9.33 (2.40), 1–15	9.01 (2.04), 4–15	9.57 (2.38), 1–15	0.049
SRS social cognition	7.67 (2.54), 0–17	7.46 (2.36), 3–17	7.83 (2.67), 0–17	
SRS social motivation	7.15 (2.62), 0–19	7.37 (2.73), 2–19	6.99 (2.53), 0–17	n.s.
SRS subscale mannerisms	2.87 (3.5), 0–20	2.96 (2.74), 0–19	2.83 (3.36), 0–20	n.s.
SRS total score	44.21 (11.10), 7–103	43.76 (11.04), 24–98	44.55 (11.16), 7–103	n.s.

WISC = Wechsler Intelligence Scale, SRS = Social Responsiveness Scale; n.s. = not significant.

**Table 2:** Overview on the structural data.

	Total (N = 285) M (SD)	Male (N = 123) M (SD)	Female (N = 162) M (SD)	Significance Male vs. Female
Brain volume				
Amygdala	Left: 1698.96 (220.34), Right: 1775.37 (252.99)	Left: 1781.61 (208.57), Right: 1874.36 (237.27)	Left: 1636.08 (208.50), Right: 1700.06 (238.83)	Left: <0.001, Right: <0.001
Caudate nucleus	Left: 4100.49 (492.75), Right: 4185.45 (513.76)	Left: 4246.48 (466.01), Right: 4328.68 (505.21)	Left: 3989.44 (484.81), Right: 4076.48 (494.51)	Left: <0.001, Right: <0.001
Hippocampus	Left: 4272.93 (446.6), Right: 4336.75 (422.48)	Left: 4508.88 (392.11), Right: 4543.96 (386.77)	Left: 4093.44 (400.44), Right: 4179.12 (379.04)	Left: <0.001, Right: <0.001
Global pallidum	Left: 1775.37 (252.99), Right: 1811.54 (200.94)	Left: 2043.52 (256.73), Right: 1903.57 (178.84)	Left: 1904.02 (251.77), Right: 1741.53 (188.64)	Left: <0.001, Right: <0.001
Nucleus accumbens	Left: 676.10 (118.08), Right: 758.87 (113.16)	Left: 716.55 (112.38), Right: 802.04 (104.68)	Left: 645.33 (113.24), Right: 726.04 (108.51)	Left: <0.001, Right: <0.001
Putamen	Left: 6621.11 (687.93), Right: 6341.24 (663.80)	Left: 7044.90 (607.55), Right: 6732.77 (610.04)	Left: 6298.72 (559.63), Right: 6043.39 (537.35)	Left: <0.001, Right: <0.001
Cerebellum	Left: 58,435.23 (5751.47), Right: 59,223.91 (5691.18)	Left: 61,948.07 (4691.11), Right: 62,572.19 (4955.44)	Left: 55,762.88 (5005.98), Right: 56,676.75 (4840.20)	Left: <0.001, Right: <0.001
Thalamus	Left: 7353.33 (727.58), Right: 7394.39 (723.82)	Left: 7731.56 (688.02), Right: 7773.15 (667.40)	Left: 7065.60 (618.35), Right: 7106.26 (627.22)	Left: <0.001, Right: <0.001
Cortical thickness				
Caudal middle frontal	Left: 2.67 (0.20), Right: 2.66 (0.18)	Left: 2.67 (0.22), Right: 2.66 (0.19)	Left: 2.68 (0.19), Right: 2.66 (0.16)	Left: n.s., Right: n.s.
Lateral orbital frontal	Left: 2.83 (0.19), Right: 2.82 (0.20)	Left: 2.82 (0.19), Right: 2.81 (0.21)	Left: 2.83 (0.19), Right: 2.82 (0.20)	Left: n.s., Right: n.s.
Medial orbital frontal	Left: 2.66 (0.18), Right: 2.62 (0.21)	Left: 2.66 (0.18), Right: 2.63 (0.21)	Left: 2.66 (0.19), Right: 2.62 (0.22)	Left: n.s., Right: n.s.
Rostral middle frontal	Left: 2.46 (0.15), Right: 2.45 (0.17)	Left: 2.46 (0.16), Right: 2.45 (0.18)	Left: 2.46 (0.14), Right: 2.45 (0.16)	Left: n.s., Right: n.s.
Superior frontal	Left: 2.93 (0.19), Right: 2.90 (0.18)	Left: 2.91 (0.20), Right: 2.89 (0.19)	Left: 2.95 (0.18), Right: 2.91 (0.17)	Left: n.s., Right: n.s.

n.s. = not significant.

## Data Availability

Ethical restrictions to protect participant confidentiality prevent us from making anonymised study data, publicly available. This also refers to the analysis/experimental code, and any other digital materials, where participant-related anonymised information are also included. Readers seeking access to the study data and materials should contact the corresponding author based on a formal collaboration agreement. The data and materials will be released to requestors after approval of this formal collaboration agreement by the local Ethics Committees.
